# Artificial Intelligence in Bariatric Surgery: Current Status and Future Perspectives

**DOI:** 10.1007/s11695-022-06146-1

**Published:** 2022-06-17

**Authors:** Mustafa Bektaş, Beata M. M. Reiber, Jaime Costa Pereira, George L. Burchell, Donald L. van der Peet

**Affiliations:** 1 Department of Gastrointestinal Surgery, Amsterdam UMC Location Vrije Universiteit Amsterdam, De Boelelaan 1117, 1081 HV Amsterdam, the Netherlands; 2 Department of Computer Science, Vrije Universiteit Amsterdam, De Boelelaan 1105, 1081 HV Amsterdam, the Netherlands; 3 Medical Library Department, Amsterdam UMC Location Vrije Universiteit Amsterdam, De Boelelaan 1117, 1081 HV Amsterdam, the Netherlands

**Keywords:** Artificial intelligence, Machine learning, Deep learning, Bariatric surgery

## Abstract

**Background:**

Machine learning (ML) has been successful in several fields of healthcare, however the use of ML within bariatric surgery seems to be limited. In this systematic review, an overview of ML applications within bariatric surgery is provided.

**Methods:**

The databases PubMed, EMBASE, Cochrane, and Web of Science were searched for articles describing ML in bariatric surgery. The Cochrane risk of bias tool and the PROBAST tool were used to evaluate the methodological quality of included studies.

**Results:**

The majority of applied ML algorithms predicted postoperative complications and weight loss with accuracies up to 98%.

**Conclusions:**

In conclusion, ML algorithms have shown promising capabilities in the prediction of surgical outcomes after bariatric surgery. Nevertheless, the clinical introduction of ML is dependent upon the external validation of ML.

## Introduction 

Artificial intelligence (AI) is a new field in medicine gaining major interest within healthcare, but its development in clinical settings is already referred to as a digital revolution for healthcare [1].

Artificial intelligence is defined as computer science capable of imitating several aspects of human intelligence and behavior [2]. With the use of large datasets, AI models can be trained to conduct several complicated tasks [3]. Machine learning, one of the domains of AI, is a computer system in which models are trained to form new predictions or decisions by analyzing large quantities of data [4]. A specific subclass of machine learning known as deep learning uses multiple layers to analyze imported data. In each layer, weights are calculated for several factors from the data. After repeating this process, a final model is trained and ready to be applied on new data. Examples of both machine and deep learning techniques are presented in Table 1.

**Table 1:** Definitions of subclasses within AI

Subclass	Definition
Machine learning (ML)	ML involves computer science that is able to perform desired tasks based on input data. When provided with sufficient data, algorithms can recognize patterns in data and train the model to perform better. After completion of the final model, the algorithm can be applied to new unknown data [5]
Decision tree (DT)	Within a DT model, multiple factors are classified into tree branches. Based on the algorithm, these branches are divided into nodes, forming several tree pathways. In the end, this model tends to find the smallest tree that optimally fits the data [6]
Gradient boosting (GBM)	In GBM, weights are added to several factors after classification. Afterwards an assessment of weights occurs, in which weights are modified based on the difficulty to classify the factors. this process is repeated until a final optimal model is generated [7]
Random forest (RF)	RF involves the formation of multiple decision trees with specific values for predictors. This technique combines all decision trees in order to build an accurate model for predictions [8]
Support vector machine (SVM)	SVM models use mapped input data to discover the optimal boundary to separate several classes and values [9]
Deep learning	As a specific branch of machine learning, deep learning can recognize patterns within datasets by using multiple processing layers. Within each layer, weights are present for several factors within the model. After the training process, an optimal model is built to perform on new data [10]
Artificial neural networks (ANNs)	Similar to our brain system, data is passed through multiple processing layers within ANNs. Each layer contains weights in order to make decisions for the resulting output. By repeat of this process, this model can improve results and produce the most accurate model in the end [11]
Convolutional neural networks (CNNs)	CNNs are a specific type of neural networks, however no weights are used in the layers. Instead, multiple layers are functioning as filters to register patterns or regions of images [12]
Radiomics	A radiomics model analyzes images in order to retrieve specific texture features that are registered as a 0 or 1. By detecting these features, various pathologies could be recognized [13]

*Abbreviations: ML*, machine learning; *DT*, decision tree; *GBM*, gradient boosting machine; *RF*, random forest; *SVM*, support vector machine; *ANN*, artificial neural networks; *CNN*, convolutional neural networks

Several potentials of AI models have already been demonstrated in clinical practice [14, 15]. For example, machine learning algorithms have been applied to MRI, X-ray, and CT images to detect tumors in various organs. Additionally, input from large numbers of electronic health records enabled AI models to identify risk factors for multifactorial outcomes such as length of stay, mortality, and early hospital readmission after surgery [16]. Recently, in colorectal surgery, machine learning was used to predict outcomes such as lymph node metastasis, response to chemoradiotherapy, and postoperative complications. For these outcomes, predictions were performed with accuracies up to 96%. This could emphasize the potential of machine learning to support risk stratification and facilitate clinical decision-making for general surgeons [17–19].

Currently, bariatric surgery has evolved to being a key in treating the worldwide pandemic of morbid obesity. Optimal postoperative weight loss including resolution of obesity-related comorbidities leads to a decreased burden of disease and related mortality [20, 21]. Despite an increasing amount of large data set studies in bariatric surgery, several factors such as short- and long-term complication rates and weight loss remain unpredictable. An example in which AI could benefit bariatric surgery is insufficient weight loss after surgery. Ten to thirty percent of patients show insufficient weight loss after bariatric surgery [22]. Risk factors for this are extremely diverse varying from socio-economic factors such as insurance policy to a specific type of microbiome [23, 24]. A complete overview of all risk factors and ideally an algorithm to calculate the risk of insufficient weight loss for each patient separately is still missing. Assembling an algorithm to identify both patients at major risk of insufficient weight loss and high risk of postoperative complications would assist the bariatric surgeon as well as the patient to reach a well-informed decision.

Despite the potential benefits of AI, the scope of machine learning applications is rarely reported. Therefore, this systematic review aims to provide an extensive overview of (potential) machine learning applications within bariatric surgery.

## Materials and Methods

### Search Strategy

A systematic search was performed in accordance with the *Cochrane Handbook for Systematic Reviews of Interventions* version 6.0 and PRISMA guidelines. To identify all relevant publications, systematic searches were conducted in the bibliographic databases PubMed, Embase.com, Clarivate Analytics/Web of Science Core Collection, and the Wiley/Cochrane Library from inception up to the 7^th^ of July 2021. The search included keywords and free text terms for (synonyms of) ‘machine learning’ combined with (synonyms of) ‘digestive system surgical procedures’ and ‘bariatric surgery’. The full search strategy can be found in the Supplementary information (see Appendix).

### Selection Process

Two reviewers (MB and JCP) conducted the title and abstract screening independently in accordance with the inclusion and exclusion criteria. Studies were only selected for full-text assessment if both reviewers agreed on inclusion. Controversies between reviewers were resolved by discussions, resulting in consensus. Studies were included if they met the following criteria: (i) describing machine learning algorithms within bariatric surgery, (ii) clinical study, (iii) including adults. Studies were excluded if they (i) did not describe bariatric surgery specifically, (ii) were not written in English, (iii) were certain publication types: reviews, editorials, letters, legal cases, or interviews.

### Risk of Bias Evaluation

The ROBINS-I assessment tool was applied by two reviewers (MB and JCP) to evaluate the methodological quality of included non-randomized studies [25]. Additionally, the PROBAST tool was used by two reviewers (MB and JCP) to assess the quality of machine learning models [26]. Conflicts between reviewers were solved by discussions.

### Data Synthesis and Outcome Assessment

Following full-text screening, the following data were extracted from the included studies; first author, year of publication, country, number of patients included, mean age of the study population, percentage of female patients, study design, follow-up time, surgical procedure, type of machine learning, external validation, purpose of machine learning, outcome measurements, and prediction performance. The categorization of studies was based on machine learning purposes and results were demonstrated separately.

## Results

### Study Selection and Characteristics

**Fig. 1:**
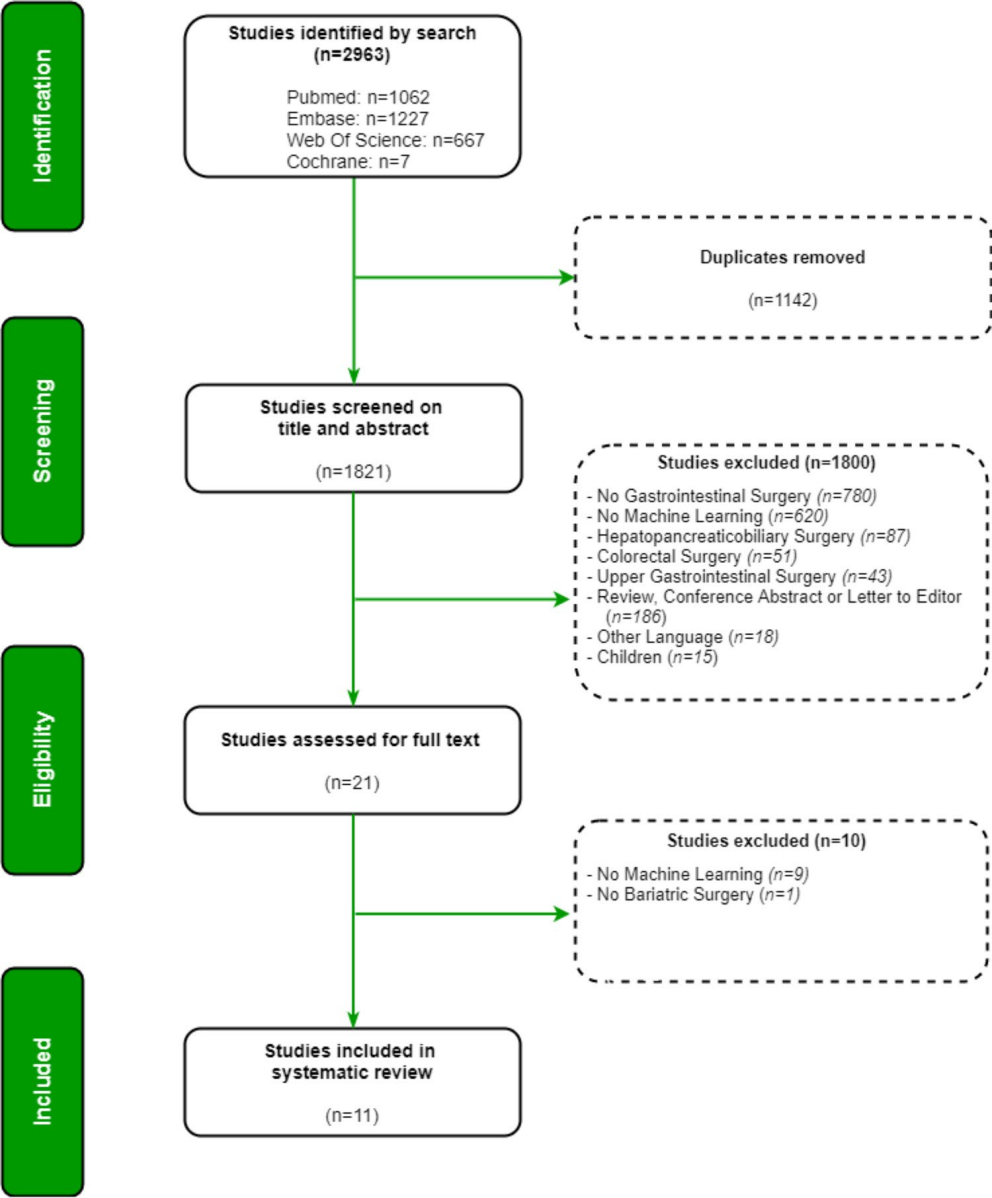
PRISMA flow diagram of the search

The systematic literature search generated a total of 1821 references after removal of duplicates**.** After screening of titles and abstracts, 21 studies remained for full-text assessment. Eleven full texts were included. The flow chart of the search and selection process is presented in Fig. 1. Table 2 summarizes the general characteristics of the included studies.

**Table 2:** General characteristics of included studies

Authors	Year	Country	Patients s	Age (mean)	Female (%)	Study design	Follow-up	Surgical procedures	Type of machine learning	External validation	ML Purpose	Study outcomes	Prediction performance (ACC/AUC)
Sheikhtaheri et al	2019	Iran	1509	39	NS	Retrospective Cohort	30 days	OAGB	Neural network	Yes	Predict postoperative complications	Accuracy; AUC	0.98/0.97
Cao et al	2019	Sweden	37,811	41	75,9	Retrospective Cohort	30 days	NS	Multiple machine learning	No	Predict postoperative complications	AUC	NA
Cao et al	2020	Sweden	44,061	42	NS	Retrospective Cohort	30 days	NS	Neural network	No	Predict postoperative complications	Accuracy; AUC	0.95/0.57
Nudel et al	2021	USA	436,807	45	79,3	Retrospective Cohort	30 days	Lap gastric bypass; LSG	Multiple machine learning	No	Predict postoperative complications	AUC	-/0.69
Wise et al	2020	USA	101,721	44	79,4	Retrospective Cohort	30 days	LSG	Neural network	No	Predict postoperative complications	AUC	-/0.59
Piaggi et al	2010	Italy	235	42	100	Retrospective Cohort	2 years	Gastric Banding	Neural network	No	Predict weight loss	AUC	-/0.80
Wise et al	2016	USA	647	47	79,6	Retrospective Cohort	1 year	Lap gastric bypass	Neural network	No	Predict weight loss	AUC	-/0.83
Lee et al	2007	Taiwan	249	33	71,1	Prospective Cohort	2 years	OAGB; Gastric Banding	Neural network	No	Predict weight loss	Accuracy	0.94/-
Aminian et al	2020	USA	13,722	54	65	Retrospective Cohort	4 years	Lap gastric bypass; LSG; Gastric Banding; Duodenal Switch	Random forest	No	Assist in decision-making	AUC	-/0,71
Assaf et al	2021	Israel	2482	43	62,7	Retrospective Cohort	-	LSG	Decision tree	No	Predict diagnosis of hiatal hernia	Accuracy	0.88/-
Cao et al	2019	Sweden	6687	43	77	Retrospective Cohort	5 years	Lap gastric bypass	Neural network	No	Predict postoperative Quality of Life	Mean squared error	NA

*Abbreviations: LSG*, laparoscopic sleeve gastrectomy; *Lap gastric bypass*, laparoscopic gastric bypass; *OAGB*, one-anastomosis gastric bypass; *NS*, not specified; *ACC*, accuracy; *AUC*, area under the curve; *NA*, not applicable

### Risk of Bias Evaluation

**Fig. 2:**
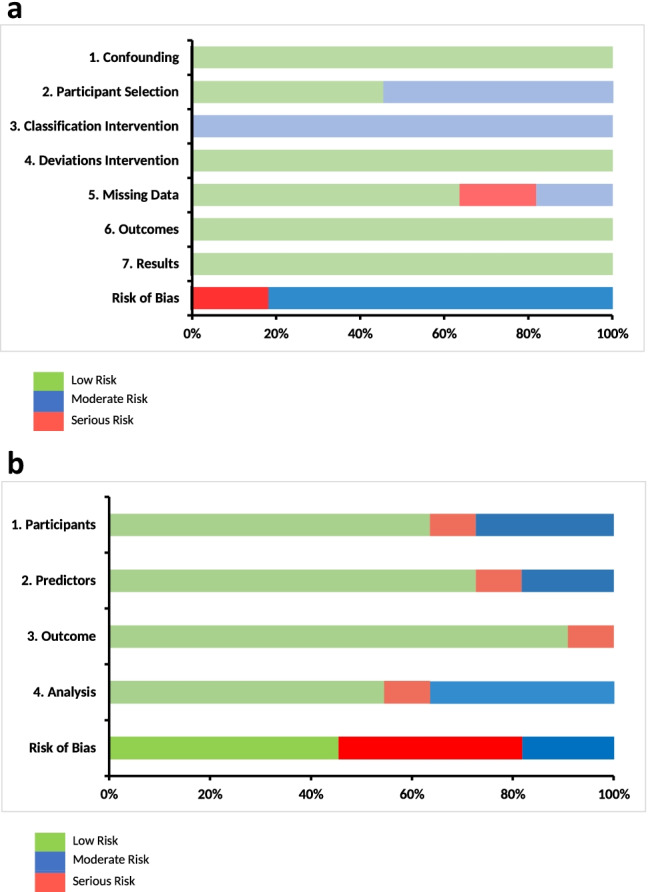
**a** Methodological quality assessment of the non-randomized studies, according to ROBINS-I assessment tool. **b** Quality of machine learning models according to the Probast tool

As all included studies were either retrospective (*n* = 10) or prospective (*n* = 1) cohort studies, the ROBINS-I assessment tool was used for quality assessment of all included studies (Fig. 2a). Since the primary outcome of this study was the type of machine learning techniques being used, domains such as bias due to confounding and bias in outcome measurements obtained low risk of bias scores. However, due to the retrospective design of these studies, a moderate risk of bias was found in the intervention classification domain. Furthermore, results of the Probast score per domain are demonstrated in Fig. 2b.

### Categorization of Machine Learning Techniques

**Fig. 3:**
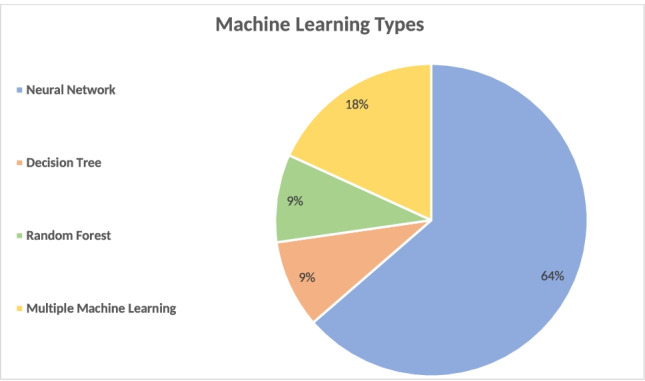
Applied forms of machine learning

Purposes of machine learning were prediction of postoperative complications (*n* = 5), prediction of the amount of postoperative weight loss (*n* = 3), aid in decision-making preoperatively (*n* = 1), predicting presence of hiatal hernias (*n* = 1), and prediction of quality of life (*n* = 1). The frequency at which each form of machine learning technique was used in the included studies is summarized in Fig 3.

#### Postoperative Complications

Five studies demonstrated the use of machine learning algorithms to predict postoperative complications.

Sheikhtaheri et al. developed a model to predict postoperative complications within 90 days after one anastomosis gastric bypass surgery (OAGB), by using an ANN algorithm [27]. These complications included bleeding, anastomotic leakage, obstruction, intraabdominal abscess, and pulmonary embolism. Thirty-two factors ranging from age and BMI to smoking and laboratory test results were considered important in this prediction model. For the postoperative period of 10 days, the highest accuracy of the model was obtained; an AUC of 0.98 was observed.

Cao et al. (2019) applied multiple machine learning algorithms to detect severe complications within 30 days after bariatric surgery [28]. Machine learning techniques included decision tree, random forest, gradient boosting, SVM, and ANN models. Results have revealed the following performances for the models (accuracy, AUC): decision tree (92%, 0.5), random forest (95%, 0.51), gradient boosting (96%, 0.58), SVM (96%, 0.5), and ANN (96%, 0.54).

Consequently, Cao et al. (2020) applied ANN, and CNN models to predict serious complications within 30 days after bariatric surgery. Serious complications were defined as Clavien–Dindo classification grade 3b and higher (i.e., anastomotic leakage, organ failure, or death) [29]. For each model, the predictive performance was described by means of the accuracy, and AUC. The ANN model showed an accuracy of 84%, and an AUC of 0.54. For the CNN model, the accuracy was 95% and the AUC appeared to be 0.57 for predicting postoperative complications.

The authors of the 4^th^ study used ANN and GBM models to predict gastrointestinal leak and venous thromboembolism in patients undergoing a laparoscopic gastric bypass or laparoscopic sleeve gastrectomy [30]. For gastrointestinal leakage, the ANN and GBM model showed the following predictive capabilities, respectively; an AUC of 0.75 and 0.70. In predicting venous thromboembolisms, the ANN algorithm and gradient boosting model achieved the following values, respectively; an AUC of 0.65 and 0.67. Out of 37 variables, the most important factors in predicting both gastrointestinal leakage and venous thromboembolisms were age, height, and weight-related measures, hematocrit, albumin, and assistant training level. A history of deep vein thrombosis was an additional important variable for prediction of venous thromboembolisms.

Wise et al. (2019) aimed to predict the readmission rate of 3.1%, the reoperation and reintervention rate of 8.7%, and the mortality rate of 0.07% within 30 days after laparoscopic sleeve gastrectomy in a large cohort [31]. For this ANN model, an AUC of 0.59 was detected. Moreover, the following seven factors appeared to be important for the prediction of 30-day morbidity and mortality: age, race, BMI, hypertension, diabetes mellitus, functional status, and previous surgery.

#### Weight Loss

All three studies aimed to predict postoperative weight loss by applying ANN models.

Piaggi et al. aimed to predict the percentage excess weight loss (%EWL) in women with severe obesity, 2 years after the laparoscopic adjustable gastric banding procedure [32]. %EWL at 2 years postoperatively was 48.2%. The ANN model developed was based on preoperative data including the comprehensive test of psychopathology Minnesota Multiphasic Personality Inventory-2. The model showed an AUC of 0.80 for this prediction. Age, paranoia, antisocial practices, and Type A behavior were independent predictors of %EWL.

Wise et al. (2016) used an ANN model to predict the percentage excess body mass index loss (%EBMIL) 180 and 360 days after laparoscopic Roux-en-Y gastric bypass surgery based on preoperative variables such as BMI, race, and gender [33]. The %EBMIL was 73.5% 1 year, postoperatively. The AUC for this model was observed to be 0.83. The variables gender, race, BMI, and diabetes mellitus appeared to be the key factors for postoperative weight loss.

Lastly, Lee et al. used 17 preoperative factors to predict successful %EWL 2 years after laparoscopic OAGB or gastric banding [34]. Success in %EWL was defined as %EWL > 50% which was accomplished by 84% of the patients. The ANN model showed an accuracy of 94% and the type of operation, HbA1c, and triglyceride levels appeared to be essential for predicting successful %EWL at 2 years postoperatively.

#### Decision-Making

Aminian et al. developed a prediction model using an RF algorithm to estimate the risk of long-term end-organ complications in patients with type 2 diabetes and obesity when considering bariatric surgery [35]. The discriminating ability at 10 years was measured in the area under the curve (AUC) and resulted in the following for the surgical and non-surgical groups, respectively; all-cause mortality 0.79 and 0.81, coronary artery events 0.66 and 0.67, heart failure 0.73 and 0.75, and nephropathy 0.73 and 0.76. The five most important variables in the prediction models of all-cause mortality were age, BMI at enrollment, history of heart failure, insulin use, and smoking status.

#### Diagnosis

Assaf et al. developed a decision tree model for the preoperative prediction of the presence of hiatal hernias (HH) in patients undergoing a laparoscopic sleeve gastrectomy procedure [36]. This is relevant as the presence of a hiatal hernia may impose per-operative technical challenges which is why foreknowledge is beneficial. The model showed an accuracy of 88.2% for the prediction of hiatal hernias. Thirteen variables were observed to be influencing the prediction of hiatal hernias, in which reflux symptoms, higher age, and BMI were discovered to be associated with a higher risk of hiatal hernias. Additionally, lower age and BMI have been discovered to be related to shorter operation lengths.

#### Postoperative Quality of Life

Cao et al. (2019) built a CNN model to predict the postoperative health-related quality of life 1, 2, and 5 years after a primary gastric bypass procedure [37]. The postoperative quality of life was measured by the RAND-SF-36 questionnaire and the obesity-related problems scale (OP). Performance of the machine learning algorithm was presented as the mean squared error, indicating the discrepancy between the observed value and predicted value. The mean squared error for the CNN model was 0.035 in predicting the postoperative quality of life.

## Discussion

From this systematic review, it can be concluded that artificial intelligence has potentials in several fields within bariatric surgery. Various models have been created to predict severe complications with AUCs up to 0.98. Secondly, weight loss was predicted by AUCs ranging from 0.80 to 0.83. Lastly, an AUC up to 0.81 was observed in predicting the postoperative quality of life, diagnosis, and end-organ complications of patients with morbid obesity.

Five studies have applied machine learning models to predict postoperative complications for patients undergoing bariatric surgery. Among several models, neural networks have shown the highest accuracy of 98% in predicting postoperative complications. Ideally, by using machine learning models, bariatric surgeons will be able to better predict (severe) postoperative complications for each unique patient. These predictions can, in theory, influence the decision towards a different type of bariatric operation or different timing of the operation, more specific prophylactic measures to prevent a certain type of complication, or a shared decision with complete informed consent.

In a recent study, the “low-risk bariatric patient” was defined by the absence of factors such as a medical history of thromboembolic events, diabetes mellitus, and kidney or pulmonary disease [38]. In this review, overlapping risk factors have been identified in the included studies predicting postoperative complications and weight loss (Table 3). It is of no surprise that age, BMI, previous intra-abdominal surgery, diabetes, and cardiovascular disease were identified as risk factors for postoperative severe complications. However, other factors such as race, inflammatory bowel disease, laboratory results, and functional status are more controversial. Not all clinical variables were included in a similar or homogeneous manner across the included studies. This is despite the hypothesis that inclusion of previously excluded variables may improve the accuracy of machine learning models to predict postoperative complications and related risk factors. In the field of breast cancer surgery, the exclusion of variables in machine learning models was prevented by determining many variables based on pre-operative, intra-operative, and post-operative means [39]. These findings could suggest that guidelines are needed to secure a comprehensive list of clinical factors that can be used for an optimal training process of machine learning models.

**Table 3:** Summary of overlapping factors for postoperative complications and weight loss

Postoperative complications		Postoperative weight loss	
Protective factors	Risk factors	Helping factors	Inhibiting factors
Low BMI	Non-White race	Female gender	Older age
	Diabetes mellitus*		Diabetes mellitus*
	Older age Previous bariatric surgery		High BMI

*BMI*, body mass index

* = type not specified

Three studies have attempted to predict postoperative weight loss. Neural networks demonstrated the highest AUC of 0.94 in predicting postoperative weight loss. For decades now, researchers in the bariatric field have attempted to identify all risk factors for insufficient weight loss after bariatric surgery. Multiple studies have shown that postoperative weight loss is dependent on multiple factors, both objective measures such as BMI and subjective measures such as patient-related measures. It could therefore be specifically beneficial and interesting for bariatric surgeons to implement AI as a means of identifying risk factors for, for example, insufficient WL. However, as Nudel et al. noted [30], external validation of the machine learning model was missing due to insufficient data. Therefore, more large datasets are needed before accurate and valid models can be developed.

For predicting the risk of long-term end-organ complications, such as coronary artery events, heart failure, and nephropathy in patients suffering from type 2 diabetes and morbid obesity, a random forest model showed an AUC of 0.66, 0.73, and 0.73, respectively. According to Aminian et al. [35], this random forest model may support and accelerate the process of decision-making toward bariatric surgery. This is desirable as the duration of obesity itself and the presence of its related comorbidities have repeatedly been reported to lead to less postoperative weight loss and higher comorbidity-related mortality [40–42]. As weight loss after bariatric surgery is not always associated with health-related quality of life, predicting the increase in quality of life after bariatric surgery is a welcome algorithm in the process of expectation management and shared decision-making, preoperatively [43, 44]. Neural networks have shown a mean squared error of 0.035 in predicting the postoperative health-related quality of life 1, 2, and 5 years after bariatric surgery, indicating an accurate estimation, since the mean squared error was close to 0. This neural network model might provide the opportunity to improve postoperative care and rehabilitation for patients undergoing bariatric surgery. However, due to missing patient information, the generalizability of this model might be uncertain. Missing data could be solved by imputation, as this was done in the study of Tseng et al. [45], in which machine learning models were used to predict acute kidney injury after cardiac surgery. One study predicted the presence of hiatal hernias. The importance of hiatal hernia (HH) present at the time of bariatric surgery remains controversial but is increasingly recommended to be corrected simultaneously with the laparoscopic sleeve gastrectomy [46]. Nevertheless, gastroesophageal reflux symptoms may worsen or persist, and a secondary operation with conversion from sleeve gastrectomy to LRYGB may be necessary [47]. The foreknowledge of the presence of HH may both influence the patient and surgeon in decision-making towards LRYGB and predict a longer operation time. However, as the authors of this study mention, the accuracy of the models developed is not impressive and the study should be regarded as proof of concept, exploring the possibilities with AI.

Due to the missing external validation in most studies, the first step for future studies in bariatric surgery should be the inclusion of external validation cohorts to gain more generalizability of machine learning models. Afterwards, clinical trials should be conducted to facilitate the implementation of ML models within bariatric surgery. For both steps, large amounts of data are required for the training process of these models. This data could be retrieved from available patient databases or robotic surgery, eventually facilitating the training process of machine learning [48, 49].

This review has revealed that machine learning models have potentials to predict postoperative complications, weight loss, end-organ complications, quality of life, and preoperative diagnosis. After the necessary steps to improve generalizability and clinical validation, machine learning models may have a significant impact on decision-making within bariatric surgery. As machine learning models are improved and validated, surgeons could be one step closer to achieving personalized decision-making for patients undergoing bariatric procedures.

To use machine learning models for the prediction of surgical outcomes in bariatric surgery, data from laparoscopic bariatric surgery should be accessible [50]. Laparoscopic videos of bariatric procedures could be collected to serve as a training database for machine learning models. By providing accurate image navigation during surgery, anatomical landmarks and unexpected intraoperative findings such as adhesions and abdominal wall hernias could be identified efficiently by machine learning models [51]. In addition, perioperative data collected from anesthesiologists could be collected such as continuous blood pressure measures or oxygen saturation as factors possibly predicting postoperative complications. Furthermore, as robotic surgery is often performed in bariatric surgery, machine learning models could also improve the performance of robotic surgery by providing 3D mapping during surgery and evaluating surgical skills afterward [52].

This review has several limitations. External validation cohorts seem to be missing for most studies, indicating the uncertainty of machine learning models. Therefore, big data from clinical settings are required to achieve appropriate generalizability and accuracy for machine learning models [53]. Additionally, due to the presence of inconsistencies in reported accuracies and AUCs, a meta-analysis could not be conducted.

## Conclusion

In this review, promising predictive capabilities of machine learning have been discovered within bariatric surgery. Machine learning has predominantly been used for prediction of postoperative complications and weight loss. However, ML algorithms have mainly been applied to datasets without external validation. To overcome this problem, additional data from large patient databases, laparoscopic surgery, or robotic surgery should be used. By validating ML models, the clinical implementation of ML will be facilitated.

## Appendix

Table 4

Table 5

Table 6

Table 7

**Table 4:** Search strategy in PubMed

Search	Query	Results
#3	#1 AND #2	**1062**
#2	“Digestive System Surgical Procedures”[Mesh] OR “Bariatric Surgery”[Mesh] OR “Laparotomy”[Mesh] OR “Roux-en-Y”[Tiab] OR “Cholecystostom*”[Tiab] OR “Choledochostom*”[Tiab] OR “Gastroenterostom*”[Tiab] OR “Jejunoileal Bypass*”[Tiab] OR “Pancreaticojejunostom*”[Tiab] OR “Peritoneovenous Shunt”[Tiab] OR “Portoenterostom*”[Tiab] OR “Gastric Bypass*”[Tiab] OR “Appendectom*”[Tiab] OR “Cholecystectom*”[Tiab] OR “Sphincterotom*”[Tiab] OR “Colectom*”[Tiab] OR “Cecostom*”[Tiab] OR “Colostom*”[Tiab] OR “Duodenostom*”[Tiab] OR “Ileostom*”[Tiab] OR “Jejunostom*”[Tiab] OR “Esophagectom*”[Tiab] OR “Hemorrhoidectom*”[Tiab] OR “Hepatectom*”[Tiab] OR “Liver Transplant*”[Tiab] OR “Pancreas Transplant*”[Tiab] OR “Pancreatectom*”[Tiab] OR “Pancreaticoduodenectom*”[Tiab] OR “Proctectom*”[Tiab] OR “gastrectom*”[tiab] OR “Gastrostom*”[tiab] OR “Esophagoplast*”[tiab] OR “Esophagostom*”[tiab] OR “Hepatectom*”[tiab]	**489,138**
#1	“Machine Learning”[Mesh] OR “Machine Learning”[Tiab] OR “machine intelligen*”[tiab] OR “machine vision*”[tiab] OR “machine learning”[tiab] OR “transfer learning”[tiab] OR “deep learning”[tiab] OR “neural network*”[tiab] OR “support vector machine*”[tiab] OR “automatic segmentation*”[tiab] OR “Long short term memory”[tiab] OR “LSTM”[tiab] OR “supervised learning”[tiab] OR “unsupervised learning”[tiab] OR “reinforcement learning*”[tiab] OR “hierarchical learning*” [tiab] OR “Image Interpretation*”[tiab] OR “Prediction model*”[tiab] OR “image recognition”[tiab] OR “perceptron”[tiab]	**154,754**

**Table 5:** Search strategy in Embase.com

Search	Query	Results
**#5**	#3 NOT #4	**1227**
**#4**	#3 AND (‘chapter’/it OR ‘conference abstract’/it OR ‘conference paper’/it OR ‘conference review’/it OR ‘editorial’/it OR ‘erratum’/it OR ‘letter’/it OR ‘note’/it OR ‘short survey’/it OR ‘tombstone’/it)	**857**
**#3**	#1 AND #2	**2084**
**#2**	‘gastrointestinal surgery’/exp OR ‘laparotomy’/exp OR ‘biliary tract surgery’/exp OR (‘Roux-en-Y’ OR ‘Cholecystostom*’ OR ‘Choledochostom*’ OR ‘Gastroenterostom*’ OR ‘Jejunoileal Bypass*’ OR ‘Pancreaticojejunostom*’ OR ‘Peritoneovenous Shunt’ OR ‘ Portoenterostom*’ OR ‘Gastric Bypass*’ OR ‘Appendectom*’ OR ‘Cholecystectom*’ OR ‘Sphincterotom*’ OR ‘Colectom*’ OR ‘Cecostom*’ OR ‘Colostom*’ OR ‘Duodenostom*’ OR ‘Ileostom*’ OR ‘Jejunostom*’ OR ‘Esophagectom*’ OR ‘Hemorrhoidectom*’ OR ‘Hepatectom*’ OR ‘Liver Transplant*’ OR ‘Pancreas Transplant*’ OR ‘Pancreatectom*’ OR ‘Pancreaticoduodenectom*’ OR ‘Proctectom*’ OR ‘gastrectom*’ OR ‘Gastrostom*’ OR ‘Esophagoplast*’ OR ‘Esophagostom*’ OR ‘Hepatectom*’):ti,ab,kw	**720,511**
#1	‘machine learning’/exp OR (‘Machine Learning’ OR ‘machine intelligen*’ OR ‘machine vision*’ OR ‘machine learning’ OR ‘transfer learning’ OR ‘deep learning’ OR ‘neural network*’ OR ‘support vector machine*’ OR ‘automatic segmentation*’ OR ‘Long short term memory’ OR ‘LSTM’ OR ‘supervised learning’ OR ‘unsupervised learning’ OR ‘reinforcement learning*’ OR ‘hierarchical learning*’ OR ‘Image Interpretation*’ OR ‘Prediction model*’ OR ‘image recognition’ OR ‘perceptron’):ti,ab,kw	335,846

**Table 6:** Search strategy in Clarivate Analytics/Web of Science Core Collection

Search	Query	Results
#4	#1 AND #2 Refined by: [excluding] DOCUMENT TYPES: (LETTER OR MEETING ABSTRACT OR EDITORIAL MATERIAL OR CORRECTION)	**667**
#3	#1 AND #2	**747**
#2	TS = (“Roux-en-Y” OR “Cholecystostom*” OR “Choledochostom*” OR “Gastroenterostom*” OR “Jejunoileal Bypass*” OR “Pancreaticojejunostom*” OR “Peritoneovenous Shunt” OR “Portoenterostom*” OR “Gastric Bypass*” OR “Appendectom*” OR “Cholecystectom*” OR “Sphincterotom*” OR “Colectom*” OR “Cecostom*” OR “Colostom*” OR “Duodenostom*” OR “Ileostom*” OR “Jejunostom*” OR “Esophagectom*” OR “Hemorrhoidectom*” OR “Hepatectom*” OR “Liver Transplant*” OR “Pancreas Transplant*” OR “Pancreatectom*” OR “Pancreaticoduodenectom*” OR “Proctectom*” OR “gastrectom*” OR “Gastrostom*” OR “Esophagoplast*” OR “Esophagostom*” OR “Hepatectom*”)	**294,577**
#1	TS = (“Machine Learning” OR “machine intelligen*” OR “machine vision*” OR “machine learning” OR “transfer learning” OR “deep learning” OR “neural network*” OR “support vector machine*” OR “automatic segmentation*” OR “Long short term memory” OR “LSTM” OR “supervised learning” OR “unsupervised learning” OR “reinforcement learning*” OR “hierarchical learning*” OR “Image Interpretation*” OR “Prediction model*” OR “image recognition” OR “perceptron”)	**477,557**

**Table 7:** Search strategy in Wiley/Cochrane Library

Search	Query	Results
#3	#1 AND #2	**7**
#2	(“Roux en Y” OR “Cholecystostom*” OR “Choledochostom*” OR “Gastroenterostom*” OR “Jejunoileal Bypass*” OR “Pancreaticojejunostom*” OR “Peritoneovenous Shunt” OR “Portoenterostom*” OR “Gastric Bypass*” OR “Appendectom*” OR “Cholecystectom*” OR “Sphincterotom*” OR “Colectom*” OR “Cecostom*” OR “Colostom*” OR “Duodenostom*” OR “Ileostom*” OR “Jejunostom*” OR “Esophagectom*” OR “Hemorrhoidectom*” OR “Hepatectom*” OR “Liver Transplant*” OR “Pancreas Transplant*” OR “Pancreatectom*” OR “Pancreaticoduodenectom*” OR “Proctectom*” OR “gastrectom*” OR “Gastrostom*” OR “Esophagoplast*” OR “Esophagostom*” OR “Hepatectom*”):ti,ab,kw	**4113**
#1	(“Machine Learning” OR “machine intelligen*” OR “machine vision*” OR “machine learning” OR “transfer learning” OR “deep learning” OR “neural network*” OR “support vector machine*” OR “automatic segmentation*” OR “Long short term memory” OR “LSTM” OR “supervised learning” OR “unsupervised learning” OR “reinforcement learning*” OR “hierarchical learning*” OR “Image Interpretation*” OR “Prediction model*” OR “image recognition” OR “perceptron”):ti,ab,kw	**4733**
